# Complete Genome Sequence of Type Strain *Campylobacter fetus* subsp. *fetus* ATCC 27374

**DOI:** 10.1128/genomeA.01344-16

**Published:** 2016-12-15

**Authors:** Luciana M. Oliveira, Daniela M. Resende, Elaine M. S. Dorneles, Elvira C. A. Horácio, Fernanda L. Alves, Leilane O. Gonçalves, Grace S. Tavares, Ana Paula R. Stynen, Andrey P. Lage, Jeronimo C. Ruiz

**Affiliations:** a Grupo Informática de Biossistemas e Genômica, Centro de Pesquisas René Rachou (CPqRR), Fiocruz Minas, Belo Horizonte, Minas Gerais, Brazil; b Programa de Pós-Graduação em Bioinformática (ICB), Universidade Federal de Minas Gerais, Belo Horizonte, Minas Gerais, Brazil; c Programa de Pós-Graduação em Biologia Computacional e Sistemas, Instituto Oswaldo Cruz (IOC), Fiocruz, Rio de Janeiro, Brazil; d Departamento de Medicina Veterinária Preventiva, Laboratório de Bacteriologia Aplicada, Escola de Veterinária, Universidade Federal de Minas Gerais, Belo Horizonte, Minas Gerais, Brazil; e Departamento de Medicina Veterinária, Universidade Federal de Lavras, Lavras, Minas Gerais, Brazil; f Programa de Pós-Graduação em Microbiologia (ICB), Universidade Federal de Minas Gerais, Belo Horizonte, Minas Gerais, Brazil; g Programa de Pós-Graduação em Ciências da Saúde, Centro de Pesquisas René Rachou (CPqRR), Fiocruz Minas, Minas Gerais, Brazil

## Abstract

*Campylobacter fetus* subsp. *fetus* is a zoonotic bacterium important for animal and public health. The complete sequencing and annotation of the genome of the type strain *C. fetus* subsp. *fetus* ATCC 27374 are reported here.

## GENOME ANNOUNCEMENT

*Campylobacter fetus* subsp. *fetus* is a zoonotic pathogen that colonizes the intestinal and genital tract of sheep and cattle, causing infertility and abortions (1). In humans, it causes gastroenteritis and occasionally bacteremia and extraintestinal infections (2). The classification of *C. fetus* in subspecies, *C. fetus* subsp. *fetus* or *C. fetus* subsp. *venerealis,* is based on clinical features, host specificity, and phenotypic tests (3).

Nonetheless, a comparison of phenotypic identification and genomic characteristics showed some discordance, especially to *C. fetus* subsp. *fetus* (4). The use of virulence genes to differentiate the *C. fetus* subspecies has been proposed (4–6); however, the small number of *C. fetus* subsp. *fetus* genomes available for comparison precludes the identification of molecular targets with diagnosis potential and a full assessment of virulence factors that determine the specificity of infection. The complete genome of *C. fetus* subsp. *fetus* ATCC 27374 (NCTC 10842, CCUG 6823, CIP 5396, DSM 5361, LMG 6442, Mouton 1), the type strain of the species and subspecies (7), isolated from a sheep fetus brain (8), is reported here.

Sequencing was performed on an Ion Torrent system (Thermo Fisher) with 36.23-fold coverage. A total of 257,181 reads were generated from sequencing, which were adapter and quality trimmed using PRINSEQ (9). Sequences were *de novo* assembled with Velvet (10). The final assembly consists of 96 contigs and *N*_50_ contig size of 33,054 bp. The assembly is composed of 94 contigs that were aligned and ordered using the ABACAS algorithm (11) and with *C. fetus* subsp. *fetus* 04/554 (accession no. NZ_CP008808) as the reference genome. Two contigs with unknown positions in scaffolds were concatenated to the linear sequence. The open reading frames (ORFs), genes encoding ribosomal RNAs (rRNAs), transfer RNAs (tRNAs), and noncoding RNAs (ncRNAs) were identified with NCBI Prokaryotic Genome Annotation Pipeline version 3.3 (12).

Prediction of transposable elements was performed by TransposonPSI (http://transposonpsi.sourceforge.net/), Tandem Repeat Finder, and BLAST against the Repbase, Dfam, GypsyDB, Pfam, and CDD databases (13–23).

The assembled genome is composed of 1,758,333 bp, with a G+C content of 33.16% and 1,853 ORFs, including four rRNAs, 42 tRNA operons, and three ncRNAs. Gene density is 0.676 genes/kb, and the average gene size is ~889 bases/gene.

Additionally, eight proteins or domains related to transposable elements showing similarity to sequences found in *Campylobacter* spp., *Neisseria meningitidis*, and *Streptomyces albus* were predicted.

The sequenced genome will enhance the understanding of *C. fetus* subsp. *fetus* and differences compared to *C. fetus* subsp. *venerealis*, particularly in genomic plasticity, physiology, and host-pathogen interactions.

### Accession number(s).

This whole-genome shotgun project has been deposited at DDBJ/ENA/GenBank under the accession no. MKEI00000000. The version described in this paper is version MKEI01000000.
